# The HOXB4 Homeoprotein Promotes the *Ex Vivo* Enrichment of Functional Human Embryonic Stem Cell-Derived NK Cells

**DOI:** 10.1371/journal.pone.0039514

**Published:** 2012-06-27

**Authors:** Aniya Larbi, Jean-Marc Gombert, Céline Auvray, Bruno l’Homme, Aurélie Magniez, Olivier Féraud, Laure Coulombel, Alain Chapel, Maria Teresa Mitjavila-Garcia, Ali G. Turhan, Rima Haddad, Annelise Bennaceur-Griscelli

**Affiliations:** 1 Inserm UMR 935, « ESTeam Paris Sud », Stem Cell Core Facility Institut André Lwoff, University Paris Sud 11, Paul Brousse Hospital, Villejuif, France; 2 Inserm UMR 935, University of Poitiers, CHU Poitiers, Poitiers, France; 3 Inserm U1016, Institut Cochin, Paris, France; 4 Cnrs UMR 8104, Paris, France; 5 University Paris Descartes, Sorbonne Paris Cité, France; 6 IRSN, PRP-HOM, SRBE, Laboratory of Radiopathology and experimental therapies, Fontenay aux Roses, France; 7 University Paris Sud 11, Faculty of Medicine, Kremlin-Bicêtre, France; 8 AP-HP, Laboratory of Hematology, University Hospitals Paris Sud, Paul Brousse Hospital, Villejuif, France; University of Minnesota, United States of America

## Abstract

Human embryonic stem cells (hESCs) can be induced to differentiate into blood cells using either co-culture with stromal cells or following human embryoid bodies (hEBs) formation. It is now well established that the HOXB4 homeoprotein promotes the expansion of human adult hematopoietic stem cells (HSCs) but also myeloid and lymphoid progenitors. However, the role of HOXB4 in the development of hematopoietic cells from hESCs and particularly in the generation of hESC-derived NK-progenitor cells remains elusive. Based on the ability of HOXB4 to passively enter hematopoietic cells in a system that comprises a co-culture with the MS-5/SP-HOXB4 stromal cells, we provide evidence that HOXB4 delivery promotes the enrichment of hEB-derived precursors that could differentiate into fully mature and functional NK. These hEB-derived NK cells enriched by HOXB4 were characterized according to their CMH class I receptor expression, their cytotoxic arsenal, their expression of IFNγ and CD107a after stimulation and their lytic activity. Furthermore our study provides new insights into the gene expression profile of hEB-derived cells exposed to HOXB4 and shows the emergence of CD34^+^CD45RA^+^ precursors from hEBs indicating the lymphoid specification of hESC-derived hematopoietic precursors. Altogether, our results outline the effects of HOXB4 in combination with stromal cells in the development of NK cells from hESCs and suggest the potential use of HOXB4 protein for NK-cell enrichment from pluripotent stem cells.

## Introduction

Human embryonic stem cells (hESCs) represent a powerful tool to analyze the early stages of human hematopoiesis [1], [2]. Most reports on hematopoietic differentiation from hESCs have focused on erythroid, megakarocytic and myeloid pathways [3]–[15], whereas the emergence of lymphocytes, and particularly of functional Natural Killer (NK) cells from hESCs has rarely been addressed so far [16], [17]. NK cells are cellular components of the innate immune system and play critical roles in protection against pathogens and cancer [18]. New therapeutic protocols aiming at improving the treatment of leukemias and cancers may commonly comprise the use of fully mature NK cells and thus require the availability of sufficient amounts of such cells [3], [14].

In the human hematopoietic system, NK cells are traditionally defined by the lack of T, B and myeloid markers and the expression of CD56 and functional receptors mediating a complex of inhibitory and activating signals that regulate their activity. Killer-Immunoglobulin-Like-Receptor (KIR) and CD94/NKG2 (A, B and C) heterodimers are the two major receptors of Major Histocompatibility Complex (MHC) class I molecules implicated in the control of NK-cell activity. Expression of these receptors is coordinated and controlled all along the differentiation of NK cells and these characteristics have been duplicated on NK cells generated *in vitro* from hematopoietic progenitors. On the other hand, the rare works dealing with the *in vitro* differentiation of NK cells from hESCs had shown that only certain stromal cells were permissive to the expression of MHC class I receptors by the differentiating NK cells [16], [17]. Recently work in mice has shown that after *in vivo* transfer, mature NK cells from MHC class–I deficient mice could be licensed to the MHC class I of the host [19]. Therefore, the establishment of new protocols to generate functional NK cells from pluripotent stem cells with the potential to target treat malignant cells is of major interest.

The HOXB4 homeoprotein is an important regulator of hematopoietic stem cell (HSC) self-renewal and expansion [20]–[26].

Currently, ectopic expression of *HOXB4* in murine ESCs enhances their hemato-myeloid potential and allows these cells to acquire “adult-like” HSC characteristics [27]–[30]. Lentiviral-based expression of *HOXB4* in hESCs results in a growth advantage *in vitro* but fails to improve engraftment [9], [11]. Moreover, enforced expression of *HOXB4* in hESCs increases blood cell forming capacity *in vitro* and promotes HSCs development into terminally differentiated myeloid cells [11], [31]. To date, one report demonstrated that recombinant HOXB4 proteins such as tPTD-HOXB4 were able to promote hematopoietic progenitor-cell formation from hESCs [13]. Since homeoproteins behave as cell-penetrating molecules in a passive and reversible internalizing process thus rendering genetic manipulations dispensable [32], we developed a cell culture system based on the active secretion of HOXB4 protein by an engineered MS-5 mouse stromal cell line, the MS-5/SP-HOXB4 cells (SP: signal peptide). These cells secrete biologically active HOXB4 protein and permit stimulation of target cells by HOXB4. In this context, we previously demonstrated the effects of HOXB4 on the expansion of functional NK cells from human HSCs [26], [33]. Utilizing this system, we investigated in this work the effects of HOXB4 on human embryoid body (hEB)-derived NK-cell progenitor expansion and differentiation, and analyzed the expression of the activating and inhibitory receptors as well as the cytotoxic potential of the differentiated NK cells.

## Methods

### Cell Lines

hESCs from the H1 cell line (WiCell Research Institute, USA) were cultured as described [34]. Cells were maintained on a mitomycin C-inactivated mouse embryonic fibroblasts feeder layer derived from ICR (CD-1) mice (Harlan Laboratories) according to Inserm guidelines. Medium was changed daily and consisted of complete DMEM/F12 1:1 (Invitrogen, Cergy Pontoise, France) supplemented with 20% Knock Out serum replacer (Invitrogen), and 10 ng/mL recombinant human basic fibroblast growth factor (bFGF) (Invitrogen). The mouse stromal cell lines MS-5 [35], [36], MS-5/SP-HOXB4 (MS-5 transduced with a lentiviral vector containing the mouse immunoglobulin κ-chain leader sequence for protein secretion upstream of the human *HOXB4* cDNA), as well as the MS-5/enhanced green fluorescent protein (EGFP) cells (MS-5 transduced with a vector containing the *EGFP* cDNA, referred to as control) (kindly provided by Dr S. Fichelson, Cochin Institut, Paris, France) [22], [26], [33], were grown in complete alpha-MEM containing 10% FCS (Abcys).

### hEBs Formation

H1 colonies were harvested and cultured with IMDM supplemented with 15% FCS (Abcys), 1 mM L-glutamine, 1% penicillin/streptomycine, 0,1 mM β-mercaptoethanol (β-ME), 1% non essential amino acids (all from Invitrogen) and 10 ng/mL bFGF. The next day, medium was changed and replaced by the same medium without bFGF and supplemented with a cocktail of cytokines consisting in 100 ng/mL human Stem cell Factor (hSCF), 100 ng/mL human Flt3-ligand (hFlt3), 10 ng/mL human Interleukin-3 (hIL-3), 10 ng/mL human Interleukin-6 (hIl-6), 50 ng/mL human granulocyte colony stimulating factor (hG-CSF), 10 ng/mL human Bone Morphogenetic Protein-4 (hBMP4), all from Abcys and 10 ng/mL human Vascular Endothelial Growth Factor (hVEGF, Promocell, Belgium). The culture medium was changed every 4–5 days until day 19 of hEBs differentiation.

### Primary Co-cultures

hEBs at day 19 were dissociated by collagenase IV and Cell dissociation buffer enzyme-free (Invitrogen) treatment. As described, hEB-derived total cells (15*10^3^–50*10^3^ cells/cm^2^) were co-cultured during 2 additional weeks with 30-Gy pre-irradiated MS-5/SP-HOXB4 or MS-5/EGFP stromal cells in standard complete H5100 human long-term culture medium (StemCell Technologies, Grenoble, France) in T25 flasks (Fisher, Illkirch, France) [26], [33]. At the end of this period cells were counted, analyzed by flow cytometry and cultured under NK cell differentiation conditions.

### Assessment of NK-cell Differentiation Potential

Cells derived from hEBs at day 19 and from primary co-cultures with either MS-5/SP-HOXB4 or MS-5/EGFP cells were cultured under NK-cell differentiation conditions (secondary co-culture). As described, [26], [33] total cells (2*10^3^–10^4^ cells/cm^2^) were co-cultured for three weeks with unmodified MS-5 cells, in RPMI 1640, complemented with 5% FCS (StemCell Technologies), 10% human AB serum (Jack Boy, Toronto, Canada), 1 mM L-glutamine, 1% penicillin/streptomycine, 0.1 mM β-ME and human recombinant cytokines: 50 ng/mL hSCF, 50 ng/mL hFlt3-Ligand, 5 ng/mL hIL-2, 20 ng/mL hIL-15 and 20 ng/mL hIL-7 (Abcys). Half of the complete media was replaced weekly.

### Staining for Flow Cytometry

Phenotypic analyzis were performed on hEB-derived cells, primary co-culture-derived cells and NK cells derived from secondary co-culture cells. Cells were incubated for 30 minutes at 4°C in phosphate buffered saline (PBS) containing 0.1% bovine serum albumine (BSA) with the following monoclonal antibodies (mAbs): CD16-fluorescein isothiocyanate (FITC) (clone 3G8), CD13-phycoerythrin (PE) (clone SJ1D1), CD14-PE (clone RMO52), CD33-PE (clone D3HL60.251), CD34-allophycocyanin (APC) (clone 581), CD43-FITC (clone DFT1), CD45-PE (clone J.33), CD56-PE/CD3-FITC (clone NKH-1/UCHT1), CD56-APC (clone N.901), CD56- (clone N.901), CD158a,h- (clone EB6.B), CD158b1/b2, j- (clone GL183), CD158e1/e2- (clone Z27), CD158i- (clone FES172), CD159a- (clone Z199), CD335 (NKP46)- (clone Bab281) PE (all from Beckman Coulter, Villepinte, France) and, CD14- (clone M5E2), CD15- (clone HI98), CD16- (clone 3G8), CD33- (P67.6), CD64- (clone 10.1), CD94- (clone HP-3D9), Perforin- (clone δG9), Granzyme A (GzA)- (clone MOPC-21), GzB- (clone GB11), CD107a- (clone H4A3) FITC, CD45 RA- (clone HI100) PE, CD161- (clone DX12), IFNγ- (clone B27)-APC, CD336 (NKP44)- (clone p44-8.1), CD337 (NKP30)- (clone p30-15) alexafluor 647, CD56-PE/Cyanin 7 (clone B159) (all from BD Biosciences, Le Pont de Claix, France). To perform intracellular staining for HOXB4, cell supernatants were removed from 48-h co-cultures of hEBs derived total cells with MS-5/SP-HOXB4 and MS-5/EGFP and cells were subsequently fixed and permeabilized with the Fix and Perm^®^ kit (Invitrogen) according to the manufacturer’s instructions. The HOXB4 protein was detected using the I12 monoclonal antibody against HOXB4 (gift from Dr A. Gould, MRC, London, UK) and PE-conjugated secondary antibody to rat IgG2a (clone 2a 8F4, Beckman Coulter). To perform intracellular staining for Perforin, Granzyme A, Granzyme B and IFNγ, cells were fixed and permeabilized with the Cytofix/Cytoperm™ kit (BD Pharmingen) according to the manufacturer’s instructions. Isotype matched FITC-, PE- and APC-conjugated irrelevant mAbs were from BD Biosciences and Beckman Coulter. When cell surface markers were investigated, dead cells were excluded using 7AAD staining. Labelled cells were analyzed using a FACSCalibur flow cytometer or a FACScantoII flow cytometer (Beckton Dickinson) and analysed with CellQuest software, FacsDiva software or FlowJo (Tristar).

### NK-cell Stimulation (IL12/K562)

At the end of the NK permissive culture period, 20*10^3^ cells were seeded in 200 µl in 96-well round-bottomed microplates in presence of IL-12 20 ng/mL or K562 cell line (ATCC, CCL-243) [37] in a ratio of effector-target (E/T) 5 to 1 in the presence of monensine (GolgiStop, BD Pharmingen) and the anti-CD107a-FITC or the control isotype for 5 hours. CD107a is a marker of intracytoplasmic cytolytic granules, which is translocated on the cell surface as a result of effector cell degranulation. The monensine prevents the acidification of the endosomal compartment and the degradation of the FITC. Thereafter the 5 hours culture, cells were labelled with the anti-CD56-PE mAb, fixed and permeabilized with the Cytofix/Cytoperm kit (BD Pharmingen), washed with Permwash (BD Pharmingen) and stained with the anti-IFNγ-APC or the control isotype. Cytotoxic cells are considered as CD107a^+^ cells expressing or not IFN-γ staining.

### Cytotoxicity Assay

We have used the Flow based killing assay (FLOKA) [38]. Briefly, the target human K562 cells were washed with PBS resuspended at the concentration of 1*10^6^ cells/mL, and then labeled at 37°C for 10 minutes with 0.5 µM final concentration of CarboxyFluorescein diacetate succinimidyl ester (CFSE, Molecular Probe). Labeling reaction was stopped with complete RPMI media. Labeled target cells (2*10^4^) were added to 96-well U bottom culture plates along with indicated effector cells in complete RPMI media. The E/T ratio was 10∶1, 5∶1 and 1∶1. After 5 hours, cells were washed resuspended in PBS and 7AAD was added to each sample. The 7AAD staining is a marker for late cells death and the percentage of 7AAD^+^ cells in the CFSE^+^ cell gate corresponded to the cytotoxicity percentage. The spontaneous death of the target alone was inferior to 8%. The cytotoxic assays were performed in triplicate.

### RNA Extraction, Reverse Transcriptase and Real-time PCR

Total RNA was extracted from hEBs using RNeasy Mini kit (Qiagen, Hilden, Germany) and one microgram of DNase-treated RNA was transcribed into complementary DNA (cDNA) using High Capacity cDNA RT Kit (Invitrogen, Groningen, Netherlands) according to the manufacturer’s instructions. The resulting cDNA was analyzed for differential gene expression by using the Real-time quantitative PCR (Q-PCR) as described previously [39] on an Applied Biosystems 7900HT Fast Real-Time PCR System (Invitrogen, Groningen, Netherlands). Briefly, Q-PCR was performed in quadruplicate analysis of two RNA samples from hEB-derived cells co-cultures with MS-5/SP-HOXB4 or MS-5/EGFP using the Power SYBR Green PCR kt (Invitrogen, Groningen, Netherlands) with 10 ng of cDNA and 300 nM of primers in a final reaction volume of 20 µL according to the manufacturer’s instructions (Invitrogen, Groningen, Netherlands). Gene expression was calculated according to the formula: DCP target (Ct control- CT sample)/DCP reference (Ct control - Ct sample). This calculation was based on the expression ratio of a target gene versus a reference gene, Hypoxanthine-guanine phosphoribosyltransferase (HPRT) as the housekeeping gene for normalizing the samples [40]. 2-DDCT designates the variation in gene expression in hEB-derived cells co-cultured with MS-5/SP-HOXB4 relative to control hEB-derived cells co-cultured with MS-5/EGFP. Results are expressed as an x-fold increase or decrease in gene expression in hEB-derived cells co-cultured with MS-5/SP-HOXB4 compared to hEB-derived cells co-cultures with MS-5/EGFP. Reverse and forward primer sequences are shown in Table S1 [41], [42].

### Cell Expansion Analysis

The fold increase of NK cell numbers following MS-5/SP-HOXB4 and MS-5/EGFP co-culture conditions result from the absolute number of NK cells recovered per culture at the end of the experiments divided by that measured at day 0 (that correspond to NK cell numbers obtained from un-co-cultured hEB cells at day 19). The relative expansion corresponds to the fold increase of NK cell numbers following MS-5/SP-HOXB4 divided by the fold increase following MS-5/EGFP co-culture conditions.

### Statistical Analysis

Data are expressed as mean ± SEM. Statistical significance was assessed using the Student’s *t* test. Differences with *p*<0.05 were considered statistically significant.

## Results

### MS-5 Feeders Promote the Generation of hEB-derived CD34^+^CD45RA^+^ Progenitor Cells

**Figure 1:**
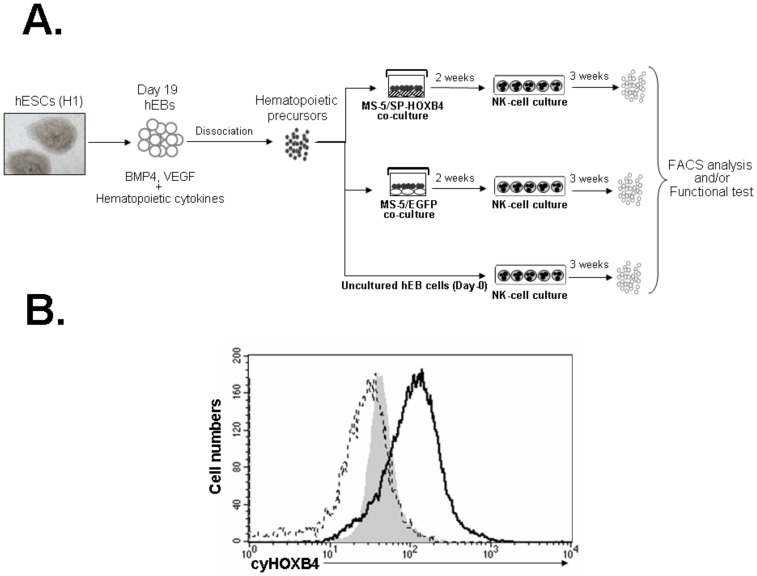
NK-cell culture procedure of hESC-derived hematopoietic precursor cells. Schematic representation of the three steps of NK-cells differentiation from hESCs. hEBs derived from the H1 hESC cell line were dissociated then co-cultured with MS-5/SP-HOXB4 cells or MS-5/EGFP cells as control during 2 weeks. Then, the cells derived from the first step of co-culture were submitted to a second step of 3-week co-culture with unmodified MS-5 cells, in permissive conditions for NK-cell differentiation in presence of SCF, IL-2 and IL-15. NK-cell culture differentiation was conducted directly with un-co-cultured hEB-derived cells as control. (B) Analysis of the presence of the HOXB4 protein within hEB-derived cells co-cultured with either MS-5/SP-HOXB4 (dark line) or MS-5/EGFP (dotted line) stromal cell lines. Data are from one experiment out of two. Gray histogram corresponds to isotypic control. Abbreviations : cy, cytoplasmic.

To assess whether passive transfer of HOXB4 protein promotes and expands hESC-derived NK-cell progenitors, we designed a two-step culture procedure as described in Figure 1A: hEB cells were first co-cultured with MS-5/SP-HOXB4 or MS-5/EGFP. Then, output cells were cultured in conditions permissive for NK-cell differentiation. We confirmed that delivery of HOXB4 in the culture supernatant leads to HOXB4 accumulation in hEB-derived cells (Figure 1B). The human genes regulated by homeoproteins during hematopoiesis are mostly unknown. However our previous work based on human CD34^+^ cells revealed that HOXB4 induces notable variations in the expression of key molecules involved in cell growth, differentiation, and transformation [42]. Thus in aim at evaluating whether HOXB4 could have a relevant effect on hEB-derived target cells and based on our previous work and those of Deneault *et al*. and Oshima *et al*. [42]–[44], we analyzed here whether HOXB4 could modulate some of its target genes in hEB-derived cells. Thus we performed gene expression profile of MS-5/SP-HOXB4 and MS-5/EGFP co-culture-derived cells regards *AML1/RUNX1*, *GATA2*, *SCL/TAL-1*, *BRCA2*, *CASP8*, *EZH2*, *GNL3*, *HPB1, MYC*, *HDAC2*, *IGFBP2* and *YPEL5*. After hEB-derived cells exposure to HOXB4, we found that *HPB1, GNL3, IGFBP2* and *HDAC2* expressions were significantly up-regulated whereas *EZH2* and *YPEL5* expressions were significantly down-regulated (Figure S1). We did not find any significant modulation of *AML1/RUNX1*, *GATA2*, *SCL/TAL-1*, *BRCA2*, *CASP8*, *IKZF* and *MYC* expressions when MS-5/SP-HOXB4 co-cultures were compared to MS-5/EGFP control co-cultures. These observations underline that HOXB4 internalization in hEB-derived cells could modulate the expression of some genes that is known to be modified by HOXB4 as previously reported [42]–[44].

We attempted to clarify the influence of HOXB4 regarding the early hematopoietic differentiation potential of hEB-derived cells. We focused on CD43 known as a good marker of very early hematopoietic precursors derived from hESCs co-cultured with OP9 stromal cells [12] and CD45RA as a marker of lymphoid specification of human CD34^+^ hematopoietic progenitors [45]–[49].

**Figure 2:**
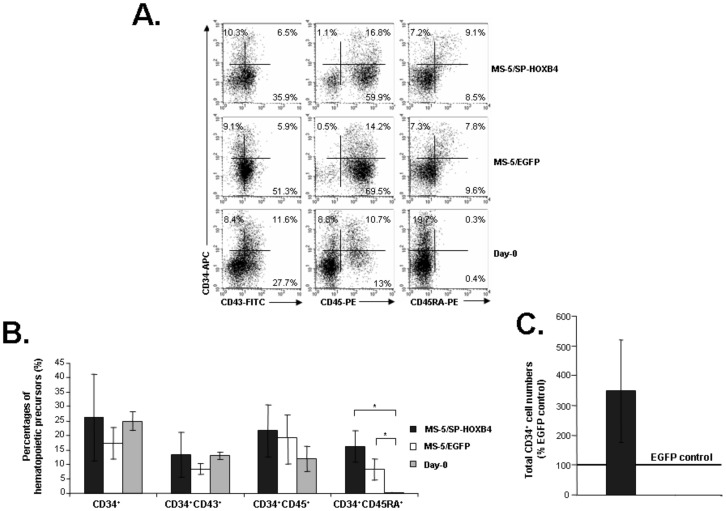
Analysis of hematopoietic progenitor cells among hEB-derived cells. (A) Phenotypic analysis of hEB-derived cells. Cells derived from un-co-cultured hEBs or from hEB cells co-cultured with MS-5/SP-HOXB4 and MS-5/EGFP, were analysed by FACS according to CD34, CD43, CD45 and CD45RA antigen expression (one representative experiment out of three). (B) Percentages of cells co-expressing CD34 and CD43, CD45 and CD45RA among total nucleated cells collected at day 14 after EB cells co-cultured with MS-5/SP-HOXB4 or MS-5/EGFP or directly obtained from un-co-cultured hEBs (n = 3, *p<0.05). (C) Relative expansion of total CD34^+^ cells. Total CD34^+^ cells were derived from hEB-derived cells co-culture with MS-5/SP-HOXB4 or MS-5/EGFP. Bar represents fold amplification relative to MS-5/EGFP control (designated as 100%) from three independent experiments.

Figure 2A illustrates the FACS analysis of one representative experiment out of five independent culture experiments. We found no significant differences using MS-5/SP-HOXB4, MS-5/EGFP or un-co-cultured hEB-derived cells in the proportions of CD34^+^ (26.3±14.9%, 17.3±5.5%, or 25±3.3%, mean ± SEM), CD34^+^CD43^+^ (13.4±7.8%, 8.5±1.9% or 13±1.4%) and CD34^+^CD45^+^ cells (21.7±9%, 19.2±7.9% or 11.9±4.3%). However, although CD34^+^CD45RA^+^ cells were generated in proportions that did not differ from both MS-5/SP-HOXB4 or MS-5/EGFP co-cultures (16.5±5.4% or 8.3±3.5%), no CD34^+^CD45RA^+^ cells could be detected from un-co-cultured hEBs (referred as day-0) (0.2±0.1%, *p*<0.05) despite an identical proportion of total CD34^+^ cells (Figure 2A and Figure 2B). These results indicate that co-culture of hEB-derived cells with MS-5 feeders with or without HOXB4 was indispensable for generation of CD34^+^CD45RA*^+^* lymphoid specified hematopoietic progenitor cells [45]–[49]. CD34^−^CD43^+^ and CD34^−^CD45^+^ cells obtained from MS-5/SP-HOXB4 and MS-5/EGFP co-cultures or from un-co-cultured hEB cells expressed myeloid markers such as CD13, CD14, CD15, CD64 or CD33 (Figure S2 and Figure S3). Interestingly CD33 was found to be expressed on a large proportion of CD34^−^CD43^+^ cells but on few CD34^−^CD45^+^ derived from MS-5/SP-HOXB4 and MS-5/EGFP co-cultures or from un-co-cultured hEB cells (Figure S2 and Figure S3). Furthermore delivery of HOXB4 in hEB-derived cells induces a 3.5±1.7 relative increase of total CD34^+^ cells (Figure 2C). This result shows a trend to expansion of hEB-derived hematopoietic precursors exposed to HOXB4.

### MS-5/SP-HOXB4 Generates Greater Numbers of NK Cells

After 19 days of hEB-cell cultures or after MS-5/SP-HOXB4 or MS-5/EGFP co-cultures of hEB-derived cells, very rare NK (CD45^+^CD56^+^CD3^−^CD19^−^) cells could be detected whereas a large proportion of CD45^+^ cells, that corresponded to myeloid cells, were recovered after MS-5/SP-HOXB4 or MS-5/EGFP co-cultures (Figures S3, Figure S4A and Figure S4B). Unselected cells derived from the primary co-cultures of hEB-derived hematopoietic progenitor cells with MS-5/SP-HOXB4, MS-5/EGFP stromal and from un-co-cultured cells (day-0 control) were then plated on unmodified MS-5 cells in conditions known to promote NK cell differentiation and maintained during three weeks.

**Figure 3:**
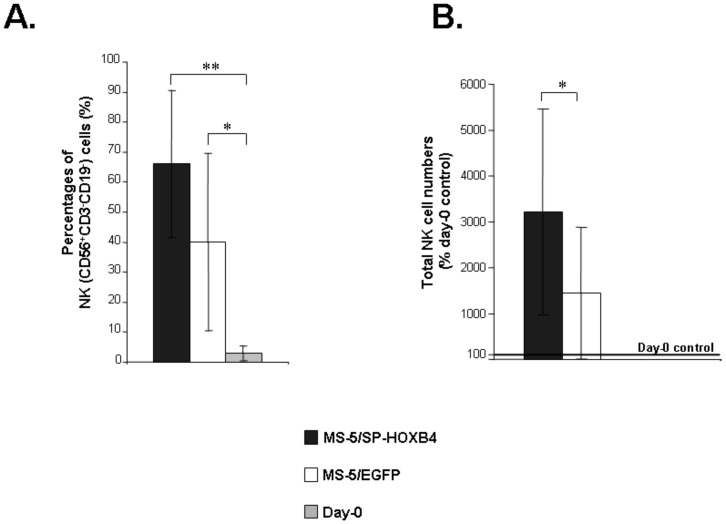
Analysis of NK differentiation potential and NK progenitor cell expansion mediated by HOXB4. (A) Percentages of NK cells (CD56^+^CD3^−^CD19^−^) among nucleated cells collected at the end of 5 weeks of co-culture. Cells derived from the 2 weeks primary co-cultures with MS-5/SP-HOXB4 or MS-5/EGFP were then plated on unmodified MS-5 cells in conditions known to promote NK-cell differentiation and maintained during three weeks. At the end of the culture period, cells were analyzed by FACS for the expression of CD56 and CD3/CD19 markers. Un-co-cultured hEB cells were directly cultured under NK-cell differentiation conditions for 3 weeks (n = 5, *p<0.05, **p<0.01). (B) Fold increase of total NK cells. NK cells were derived from total cells isolated from the primary 2-week co-cultures of hEB-derived cells with either MS-5/SP-HOXB4 or MS-5/EGFP control and then cultured under NK-cell differentiation condition for three weeks. NK cells were then numbered. Bars represent fold amplifications relative to day-0 control (un-co-cultured hEBs) (designated as 100%) (n = 5, *p<0,05).

NK-cell differentiation potential of output hEB-cells was similar in term of proportion of NK cells, *i.e*. 66.2±24.4% when cultured on MS-5/SP-HOXB4 versus 40±29.5% when cultured on MS-5/EGFP, whereas un-co-cultured hEBs generated 3±2.3% NK cells (Figure 3A). In our NK culture conditions, all CD56^+^CD3^−^CD19^−^ cells were CD45^+^ and no CD56^+^ cells could ever be detected within the rare CD45^−^ cell fractions indicating that all CD56^+^ cells that could be recovered from NK cultures correspond to hematopoietic cells (Figure S4C). Non-NK cells that could be identified among NK condition cultures corresponded to a mixture of myeloid cells (CD45^+^CD14^+^/CD15^+^/CD64^+^) (Figure S4C).

Taken together, these results show that co-culture of hEBs with MS-5 feeders with or without HOXB4 was essential for further generation of NK cells.

Moreover, in five independent experiments illustrated in Figure 3B, we found that there was a 32.2±22.3-fold mean increase in total NK-cell numbers after MS-5/SP-HOXB4 co-culture conditions whereas NK cells derived from co-cultures of hEB cells with MS-5/EGFP were expanded by only 14.3±14.5-fold (*p<*0.05) (relative improvement in NK-cell numbers: 1.7 to 14.7). The high standard deviations are due to an important degree of heterogeneity between the experiments inherent to the manipulation of hESCs. These results show that hematopoietic precursor-cells derived from co-cultures with MS-5/SP-HOXB4 generated greater numbers of NK cells than their counterparts derived from co-cultures with MS-5/EGFP (Figure 3B).

Furthermore after 2-week co-culture of hEB-derived cells with MS-5/SP-HOXB4 followed by a 3-week NK-cell differentiation culture, the absolute expansion rate of total cells (*i.e* NK cells and non-NK cells) was 0.7±0.3 with MS-5/SP-HOXB4 whereas the absolute expansion rate of total cells was 0.4±0.2 with MS-5/EGFP (Figure S5). These data suggest strongly that HOXB4 regulates the survival and/or expansion of hematopoietic precursors during cultures, since the number of cells retrieved was 2.1±0.6 times higher in the presence than in the absence of HOXB4. This result indicates that HOXB4 could enhance the final growth of total cells (that is NK cells and myeloid cells) at the end of the culture period in comparison to MS-5/EGFP cultures.

Altogether, these results show that MS-5 is mandatory for hESCs to express an NK potential, and the presence of soluble HOXB4 further acts to enhance the final output of NK cells (as well as myeloid cells). Finally, HOXB4 combined to the presence of the MS-5stromal cells line could increase the final growth of hematopoietic precursors leading to the enrichment of NK cells, in NK-cell differentiation conditions, without modifying the NK differentiation potential of hEB cells.

### NK Cells Derived from Co-cultures with MS-5/SP-HOXB4 Express Inhibitory and Activating Receptors

**Figure 4:**
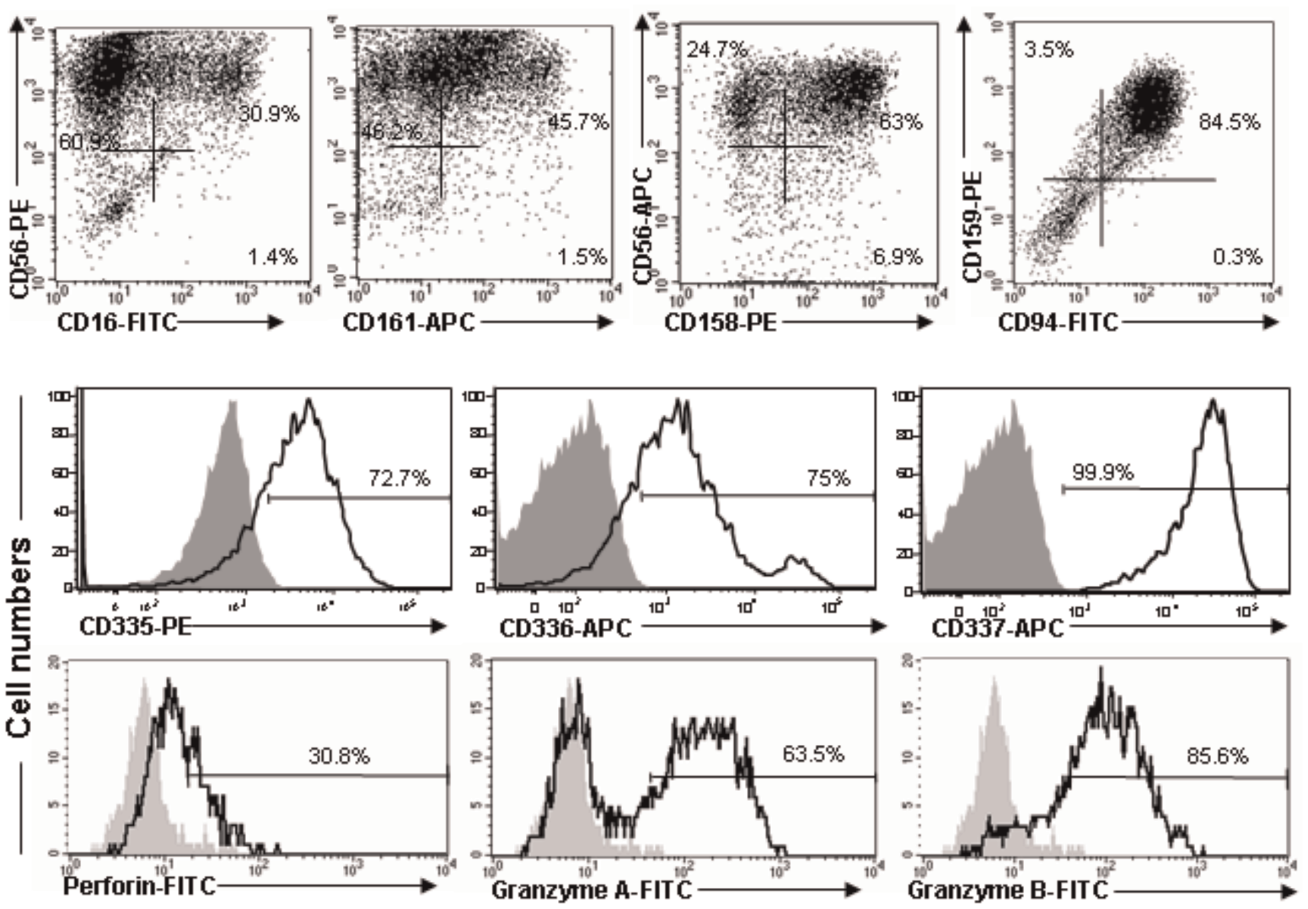
Expression of inhibitory and activating receptors and of the cytotoxic arsenal of NK cells derived from co-culture with MS-5/SP-HOXB4. NK cells were obtained from NK progenitors derived from hEB cells co-cultured with MS-5/SP-HOXB4. Cells were FACS analyzed for surface expression of CD56, CD16, CD94, the mix of CD158a, h, CD158b1/b2, j, CD158e1/e2 and CD158i (referred as CD158) and of CD159, CD335, CD336 and CD337. For CD159, CD94, CD335, CD336 and CD337 expression, cells were gated on CD56^+^ cells (not shown). NK cells obtained using the HOXB4 co-culture model were also analyzed for the intra-cytoplasmic expression of Perforin, Granzyme-A and Granzyme-B. Cells were gated on CD56^+^ cells (not shown). Data are from one experiment out of two.

**Figure 5:**
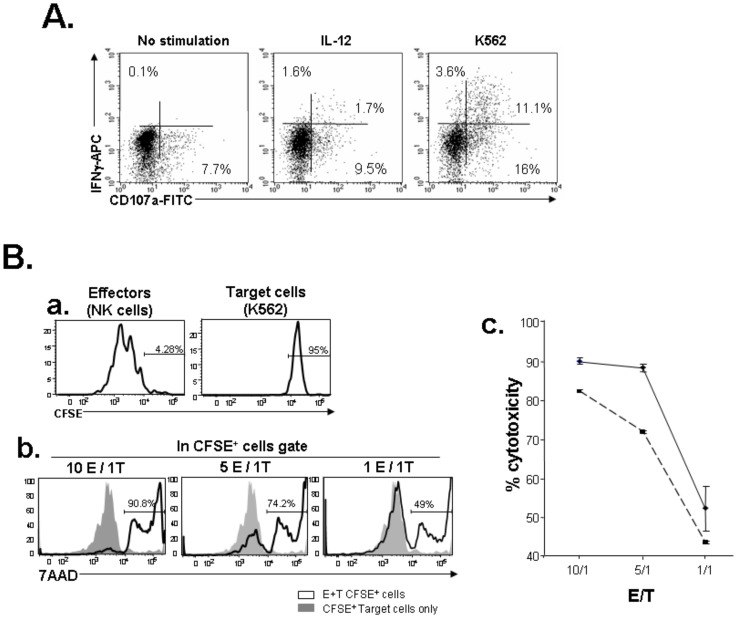
Functional activity of NK cells derived from co-culture with MS-5/SP-HOXB4. (A) The functional activity of cells was assessed in the presence of IL-12 and against K562 target cells; the NK-cell activity was evaluated by the mobilisation of the CD107a antigen on the cell surface and the intra-cytoplasmic expression of IFNγ. Cells were gated on CD56^+^ cells (not shown). (B) Cytotoxic activity of NK effector cells against target K562 cells. a) Target K562 cells were labelled by CFSE. b) Percentage of 7AAD^+^ death cells among the CFSE^+^ target cells at various Effector-Target (E/T) ratios (one experiment out of two). c) Percentage of cytotoxicity (*i.e* percentage of 7AAD^+^ death cells among the CFSE^+^ target cells) according to the ratio E/T (dotted line and bold line represent two independent experiments).

We next evaluated whether NK cells expanded by HOXB4 were endowed with functional NK properties. All NK cells expressed the dimeric inhibitory NK receptor CD94/CD159a (NKG2A) and CD159 (NKG2D) and a fraction of them expressed the Fc gamma receptor CD16, the NK receptor CD161 and the KIR proteins CD158 (CD158a, b1, b2, e1, e2, h, i, j). More than 70% of these NK differentiated cells expresses the NK cell activating receptors (natural cytotoxicity receptors) such as CD335 (NKp46), CD336 (NKp44) and CD337 (NKp30) (Figure 4). These results showed that hEB cells exposed to HOXB4 could differentiate into NK cells expressing the whole panel of maturation markers. Functionally, the majority of NK cells expressed a complete cytotoxic arsenal, with perforin, Granzyme A and Granzyme B (Figure 4). Furthermore, a fraction of NK cells exposed to K562 cells, expressed surface CD107a and intracellular IFN-γ cytokine demonstrating a fully functional phenotype. Moreover, stimulation by IL-12 also induced IFN-γ expression suggesting that hEB-derived NK cells were sensitive to this cytokine (Figure 5A) [50]. Finally, NK differentiated from hEB exposed to HOXB4 had a powerful cytotoxic activity against K562 cells in a dose dependent manner (Figure 5B) since target cells stained with CFSE are all the better killed (7AAD positive cells) as the number of NK cells effectors is important.

## Discussion

In this report, we explored the influence of the HOXB4 homeoprotein on the generation and expansion of hESC-derived NK-cell progenitors. We previously established a model in which the passive transduction of the HOXB4 homeoprotein into hematopoietic cells led to *ex vivo* expansion of human HSCs and myeloid and lymphoid progenitors in the absence of exogenously added cytokines [22], [26], [33]. We provided evidence that HOXB4 could be internalized within hEB-derived cells (Figure 1B). We show here that HOXB4 promotes the survival and/or expansion of hematopoietic precursors able to differentiate in NK cells in permissive conditions since the number of NK cells retrieved was 1.7 to 14.7 times higher in the presence of HOXB4. Thus co-culture of hEB-derived hematopoietic precursors with MS-5/SP-HOXB4 stromal cells results in a substantial output of NK cells without disturbing the intrinsic NK-cell differentiation potential of hEB-derived cells (Figure 3 and Figure S5). Finally, HOXB4 could enhance the output of hEB-derived hematopoietic precursors that are able to efficiently differentiate in NK cells under permissive culture conditions whereas MS-5 cells were required to reveal NK-cell differentiation potential of hEB-derived cells (Figure 2). Furthermore, regarding our experimental model, one may wonder which culture step allows NK-cell enrichment by HOXB4. NK-cell enrichment induces by HOXB4 could proceed from the cumulative effects of the first co-culture step that shows a trend to expansion for hEB-derived cells exposed to HOXB4 (Figure 2C) and from the secondary NK-culture condition which gives a growth advantage to hEB-derived cells (endowed with NK and myeloid differentiation potentials) exposed to HOXB4 (Figure S5) leading to a final substantial output of NK cells (Figure 3). These results give new insights regards the expansion of hESC-derived hematopoietic precursors by the HOXB4 homeoprotein and open perspectives concerning the potential effects of HOXB4 on hematopoietic differentiation from human induced pluripotent stem cells (hiPSCs) as it was recently shown in the murine model [51].

Activated NK cells could represent a potential tool for the therapy of hematopoietic malignancies combined with allogenic bone marrow transplantation [14], [52], [53]. The rare works dealing with the *in vitro* differentiation of NK cells from hESCs had shown that only certain stromal cells (S17 and AFT024) were permissive to the expression of MHC class I receptors by the differentiating NK cells [16], [17]. Here we show that contact with MS-5 cells was an absolute requirement to the expression of the intrinsic NK-cell differentiation potential (Figure 3A and Figure S4C) whereas HOXB4 appears to have a growth action (Figure 3B and Figure S5). Furthermore using H9 hESCs and two-step co-cultures with S17 and AFT024 stromal cells, Woll *et al*. generated functional NK cells that displayed potent *in vivo* anti-tumor activity [16], [17]. Here we show that using co-cultures comprising MS-5 stromal cells, HOXB4-expanded NK-cell progenitors generated from H1 hESC-derived EBs, could give rise to fully mature NK cells (Figure 4). Interestingly, HOXB4-enriched functional NK cells produce IFN-γ after stimulation by K562 targets and exhibit a powerful lytic activity against them (Figure 5) providing a new strategy for generating pluripotent stem cell-derived NK cells for potential therapeutic applications. However, in our hands, this system still less powerful than a protocol based on the HOXB4 NK-cell progenitor expansion from adult hHSCs which could allow the production of large amounts of NK cells as we showed previously [26], [33]. This might be due to hEB hematopoietic precursors-derived cells that are intrinsically different from adult hHSCs and respond less efficiently to HOXB4 than adult hHSCs do. Furthermore, regarding our experimental model, one may also wonder whether HOXB4-dependent expansion is due to the homeoprotein activity in hEB-derived cells (direct effect) or to other factors attributed to HOX-expressing MS-5 cells (indirect effect). Several observations argue strongly against the latter mechanism. First, MS-5/SP-HOXB4 cells induced enhanced human cell expansion when compared with MS-5/HOXB4 (MS-5 transduced with a lentiviral vector which does not contain the mouse immunoglobulin κ-chain leader sequence for protein secretion upstream the human *HOXB4* cDNA) indeed CD34^+^CD38^low^ cells were much less sensitive to the action of non actively secreted HOXB4, thus providing an internal control against an indirect effect of HOXB4 through MS-5 cell changes [22]. Second, the phenotype characteristics (such as morphology and growth rate) of MS-5 cells were not modified by any of the transductions as already shown [22], [26], [33], [42]. Third, MS-5 cells engineered to secrete lower amounts of human HOXB4 revealed a dose dependent effect on the response of hematopoietic precursors [22]. This strongly argues for a direct role of the secreted homeoproteins on human HSCs.

At the molecular level, the mechanisms of action of *HOX* genes during hematopoiesis remain elusive. Although reports have described the involvement of *HOX* genes in some molecular pathways in mice [54], [55], the HOX protein targets in human hematopoietic cells are mostly unidentified especially if these cells correspond to precursors derived from ESCs. However, we compared our results with published data from different hematopoietic and embryonic cell models, particularly, studies including gene expression analysis of cells that over-expressed HOXB4 [42]–[44]. Here, our observations outline that HOXB4 internalization in hEB-derived cells could modulate the expression of some genes that regulate stem cells and cancer cells, transcription, cell cycle progression, developmental events or cell differentiation (Figure S1) [42]–[44]. Gene expression variations reported in this study and in various publications often lead to divergent or contradictory results because the models used in the studies are different. Here, our molecular analysis based on a model of human ESC-derived hematopoietic precursors exposed to HOXB4, i) gives new insights into the modulation of gene expression by HOXB4, ii) might add a supplementary degree of complexity regarding potential HOXB4 target gene expressions and iii) confirms that the observed effect of HOXB4 on NK-cell production from hEB-derived cells is relevant.

Finally, our study describes for the first time the emergence of CD34^+^CD45RA^+^ cells from hESC-derived hEB cells co-cultured with MS-5 stromal which could give new perspectives regards the early steps of human lymphopoiesis from pluripotent stem cells. Indeed, regarding the early steps of human hemato-lymphopoiesis, J. Dick’s group recently provided a comprehensive analysis of the human hematopoietic hierarchy and identified human multi-lymphoid progenitors, as a distinct population of CD45RA^+^ cells in the CD34^+^CD38^−^ adult stem cell compartment, that give rise to all lymphoid cell types, as well as monocytes, macrophages and dendritic cells, which indicated that these myeloid lineages arise in early lymphoid lineage specification marked by the early acquisition of CD45RA [48], [49]. Thus based on these new insights regards human hematopoiesis and on our previous works regards early human lymphopoiesis [46], [47], CD45RA could be considered as a candidate marker for lymphoid specification of pluripotent stem cells.

Overall, these results open a field on the characterization of hESC- and iPSC-derived early lymphoid progenitors and provide experimental tools to model genetic disorders of the lymphoid system development from pluripotent stem cells. Finally, they could be the basis for the future development of immunotherapy strategies for cancer or leukemia.

## Supporting Information

Figure S1 **Gene expression modulations by HOXB4.** Gene expression changes for *AML1/RUNX1 GATA2, SCL/TAL1, BRCA2, CASP8, EZH2 GNL3, HBP1, HDAC2, IGFBP2, IKZF, YPEL5, MYC* transcripts were measured by quadruplicate analysis of two RNA samples from human hEB-derived cells co-cultured with MS-5/SP-HOXB4 or MS-5/EGFP. Relative differences in gene expression were calculated by using the 2-ddCt method, which involves normalizing the Ct value for each gene to the Ct value of the HPRT housekeeping gene. Values are shown as the fold induction in hEB-derived cells co-cultured with MS-5/SP-HOXB4 (grey bars) compared to hEB-derived cells co-cultured with MS-5/EGFP (black bars). Data represent mean ± SEM, NS: not significant, n = 4, *p<0.05, **p<0.01.
(TIF)

Figure S2 **Phenotypic analysis of CD34** ^−^ **CD43^+^ cells.** CD34^−^CD43^+^ cells derived from hEBs at day 19 of culture (day-0 control condition) and hEB-derived cells co-culture with MS-5/SP-HOXB4 or MS-5/EGFP stromal cells were FACS analyzed for the cell surface expression of myeloid markers such as CD13, CD14 and CD33. Data represent on experiment out of two.
(TIF)

Figure S3 **Phenotypic analysis of CD34** ^−^ **CD45^+^ cells.** CD34^−^CD45**^+^** cells derived from hEBs at day 19 of culture (day-0 control condition) and hEB-derived cells co-culture with MS-5/SP-HOXB4 or MS-5/EGFP stromal cells were FACS analyzed for the cell surface expression of myeloid markers such as CD14, CD15, CD33 and CD64. Data represent on experiment out of two.
(TIF)

Figure S4 **Phenotypic analysis of CD56^+^ cells.** CD56^+^ cells derived from hEBs at day 19 of culture (day-0 control condition) and hEB-derived cells co-culture with MS-5/SP-HOXB4 or MS-5/EGFP stromal cells were FACS analyzed for the cell surface expression of CD45 and CD14/15/64. Data represent on experiment out of four.
(TIF)

Figure S5 **Fold increase of total cells in NK condition.** Total cells (NK cells and non-NK cells) were derived from the primary 2-week co-cultures of hEB-derived cells with either MS-5/SP-HOXB4 or MS-5/EGFP control and then cultured under NK-cell differentiation condition for three weeks. Total cells were then numbered. Bar represents fold amplification relative to day-0 control (un-co-cultured hEBs) (designated as 100%) (n = 5, *p<0,05).
(TIF)

Table S1 **Primer sequences and access numbers**
(DOCX)
