# Supplementary figures and images for: The HOXB4 Homeoprotein Promotes the Ex Vivo Enrichment of Functional Human Embryonic Stem Cell-Derived NK Cells
Source: PLoS One. 2012 Jun 27;7(6):e39514. doi: 10.1371/journal.pone.0039514 (PMC3384663; doi:10.1371/journal.pone.0039514)

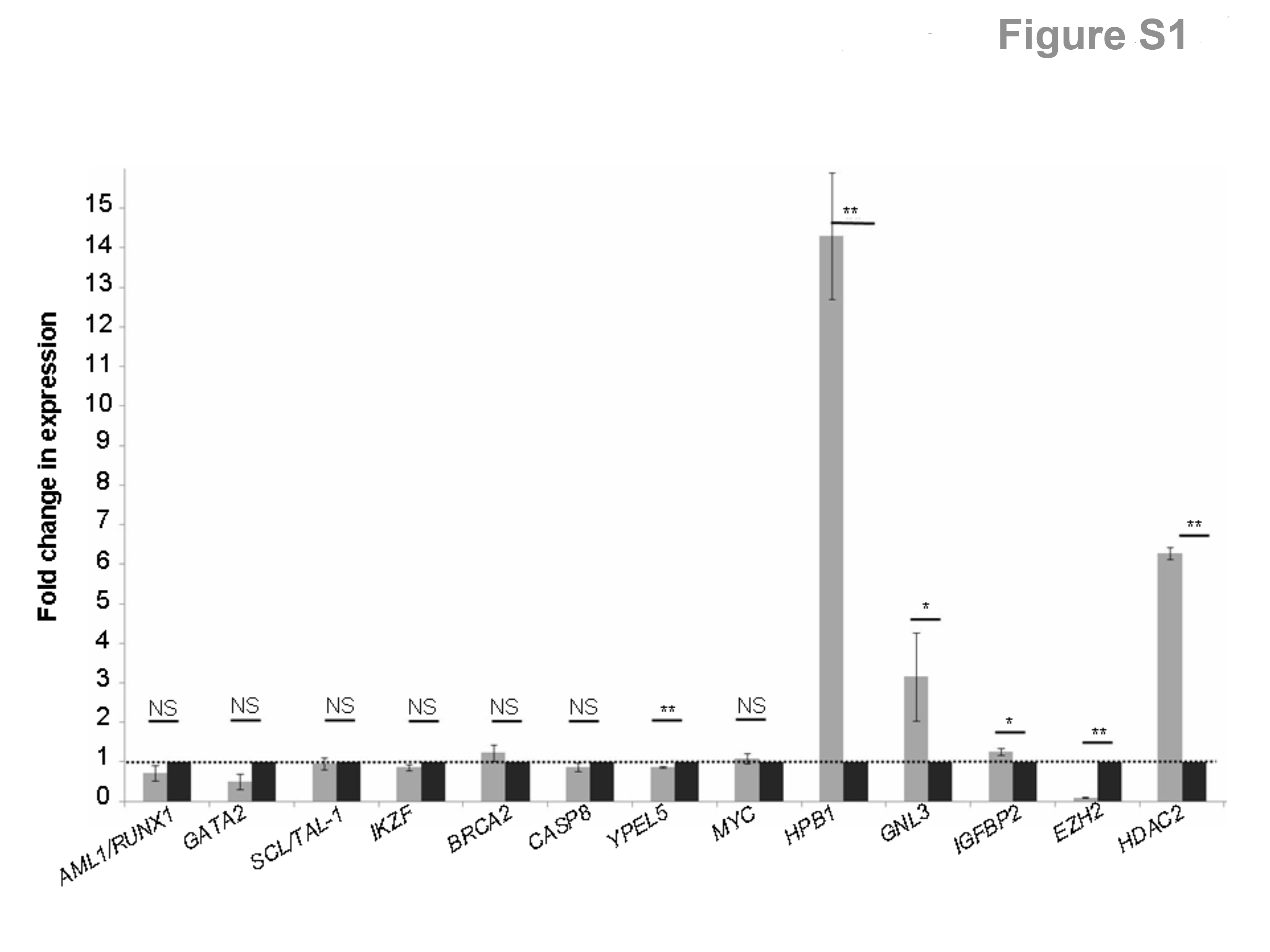

Supplement: Figure S1 — Gene expression modulations by HOXB4. Gene expression changes for AML1/RUNX1 GATA2, SCL/TAL1, BRCA2, CASP8, EZH2 GNL3, HBP1, HDAC2, IGFBP2, IKZF, YPEL5, MYC transcripts were measured by quadruplicate analysis of two RNA samples from human hEB-derived cells co-cultured with MS-5/SP-HOXB4 or MS-5/EGFP. Relative differences in gene expression were calculated by using the 2-ddCt method, which involves normalizing the Ct value for each gene to the Ct value of the HPRT housekeeping gene. Values are shown as the fold induction in hEB-derived cells co-cultured with MS-5/SP-HOXB4 (grey bars) compared to hEB-derived cells co-cultured with MS-5/EGFP (black bars). Data represent mean ± SEM, NS: not significant, n = 4, *p<0.05, **p<0.01. (TIF) [file pone.0039514.s001.tif]

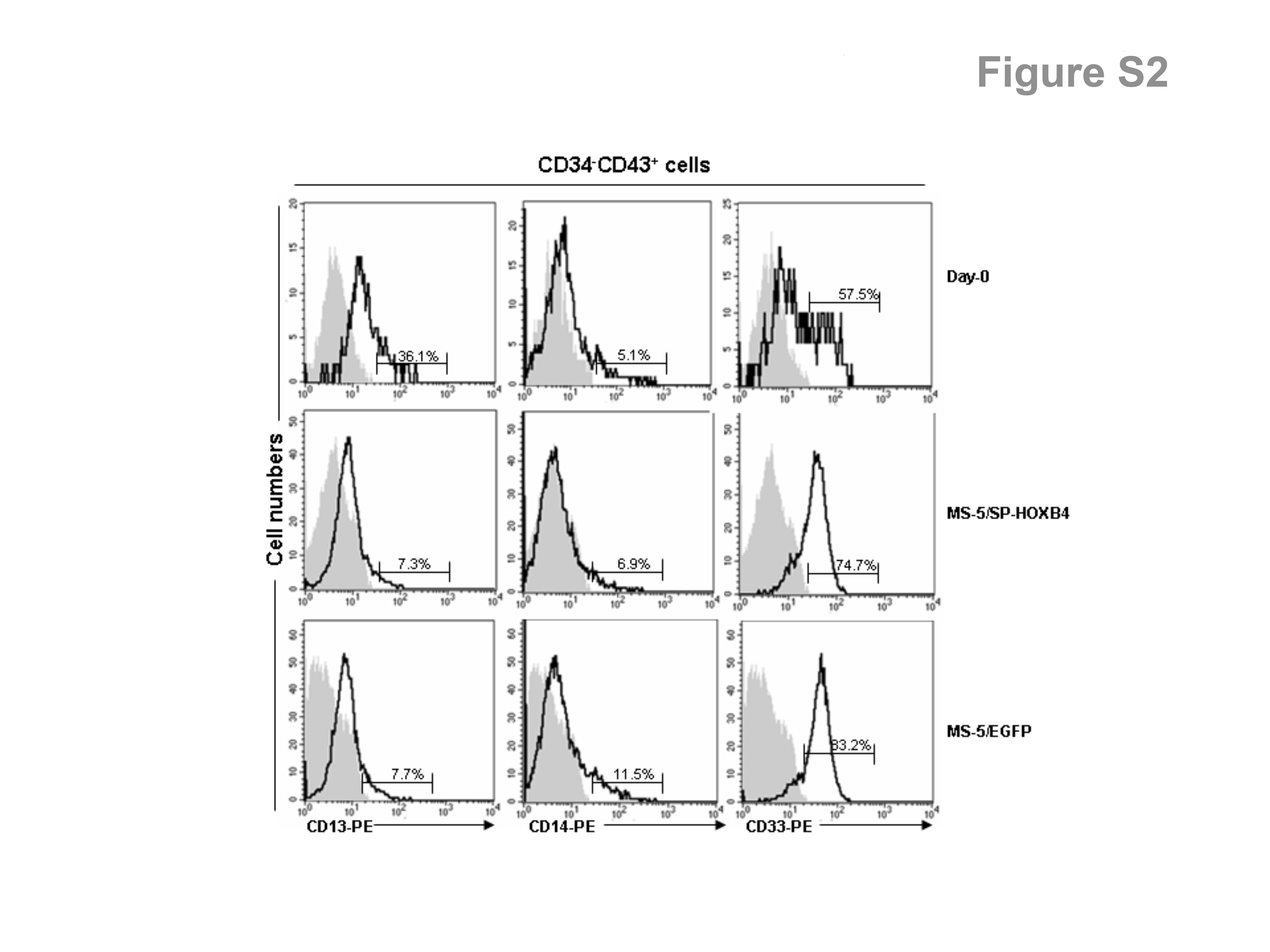

Supplement: Figure S2 — Phenotypic analysis of CD34 − CD43+ cells. CD34−CD43+ cells derived from hEBs at day 19 of culture (day-0 control condition) and hEB-derived cells co-culture with MS-5/SP-HOXB4 or MS-5/EGFP stromal cells were FACS analyzed for the cell surface expression of myeloid markers such as CD13, CD14 and CD33. Data represent on experiment out of two. (TIF) [file pone.0039514.s002.tif]

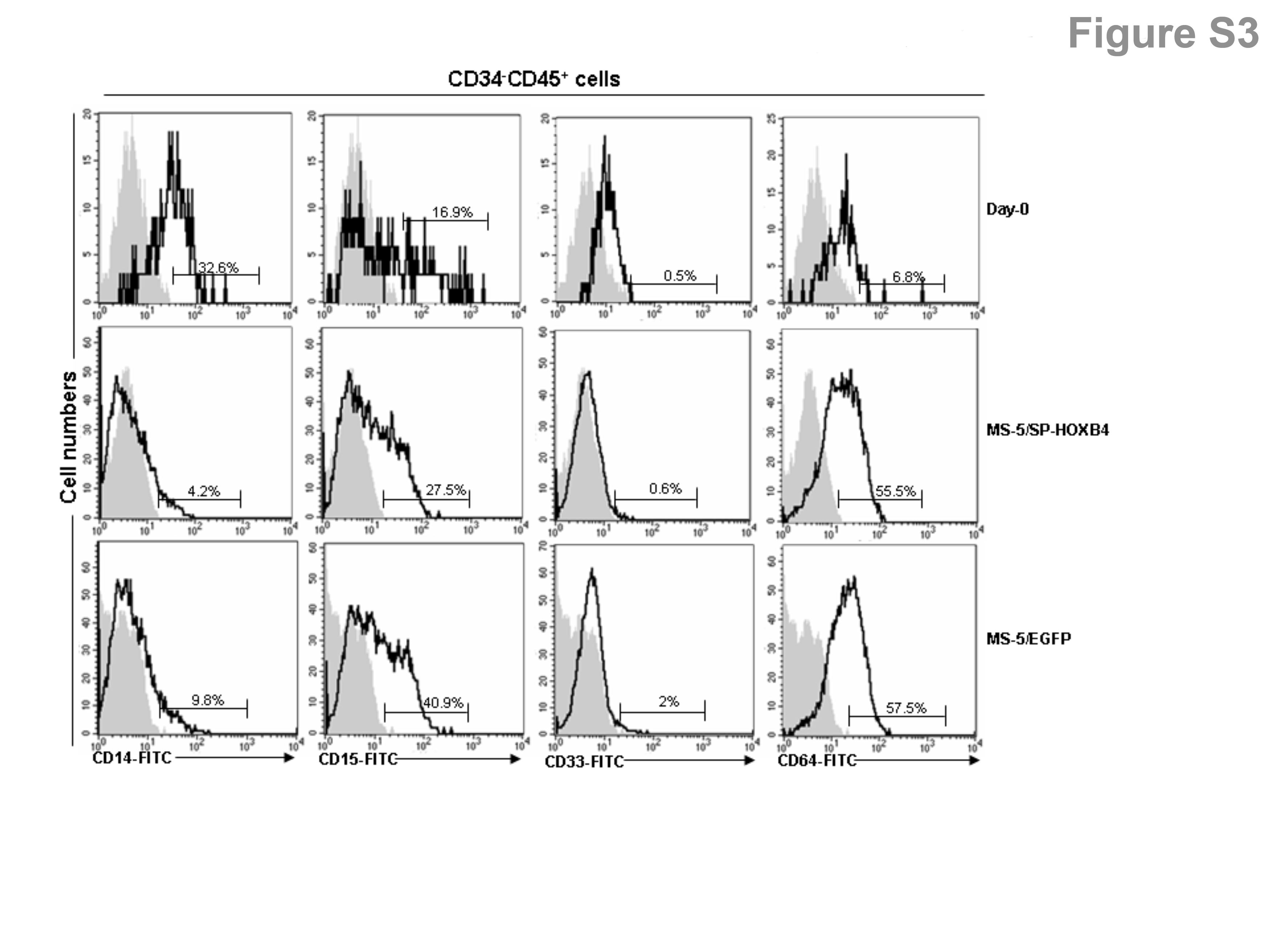

Supplement: Figure S3 — Phenotypic analysis of CD34 − CD45+ cells. CD34−CD45+ cells derived from hEBs at day 19 of culture (day-0 control condition) and hEB-derived cells co-culture with MS-5/SP-HOXB4 or MS-5/EGFP stromal cells were FACS analyzed for the cell surface expression of myeloid markers such as CD14, CD15, CD33 and CD64. Data represent on experiment out of two. (TIF) [file pone.0039514.s003.tif]

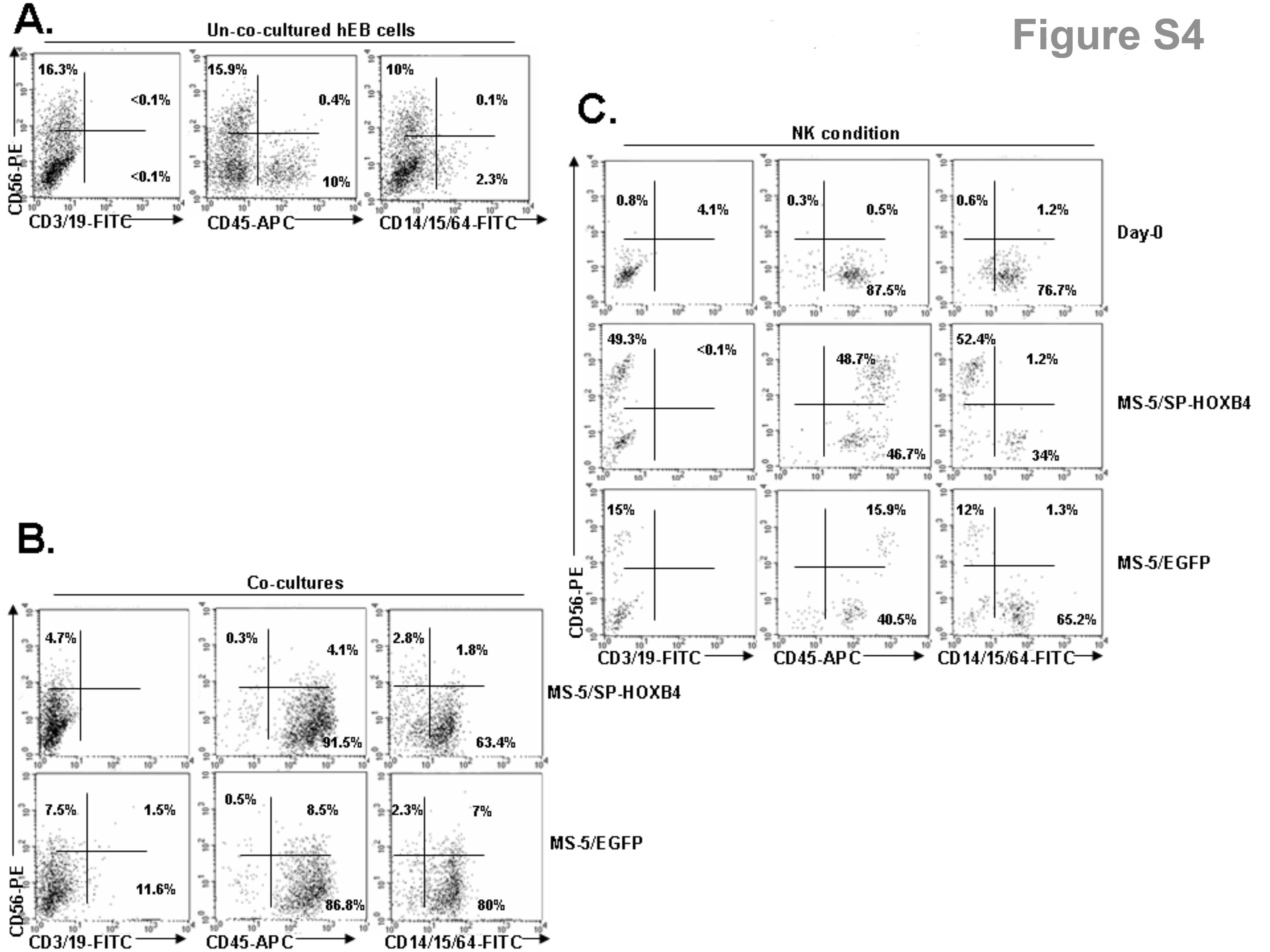

Supplement: Figure S4 — Phenotypic analysis of CD56+ cells. CD56+ cells derived from hEBs at day 19 of culture (day-0 control condition) and hEB-derived cells co-culture with MS-5/SP-HOXB4 or MS-5/EGFP stromal cells were FACS analyzed for the cell surface expression of CD45 and CD14/15/64. Data represent on experiment out of four. (TIF) [file pone.0039514.s004.tif]

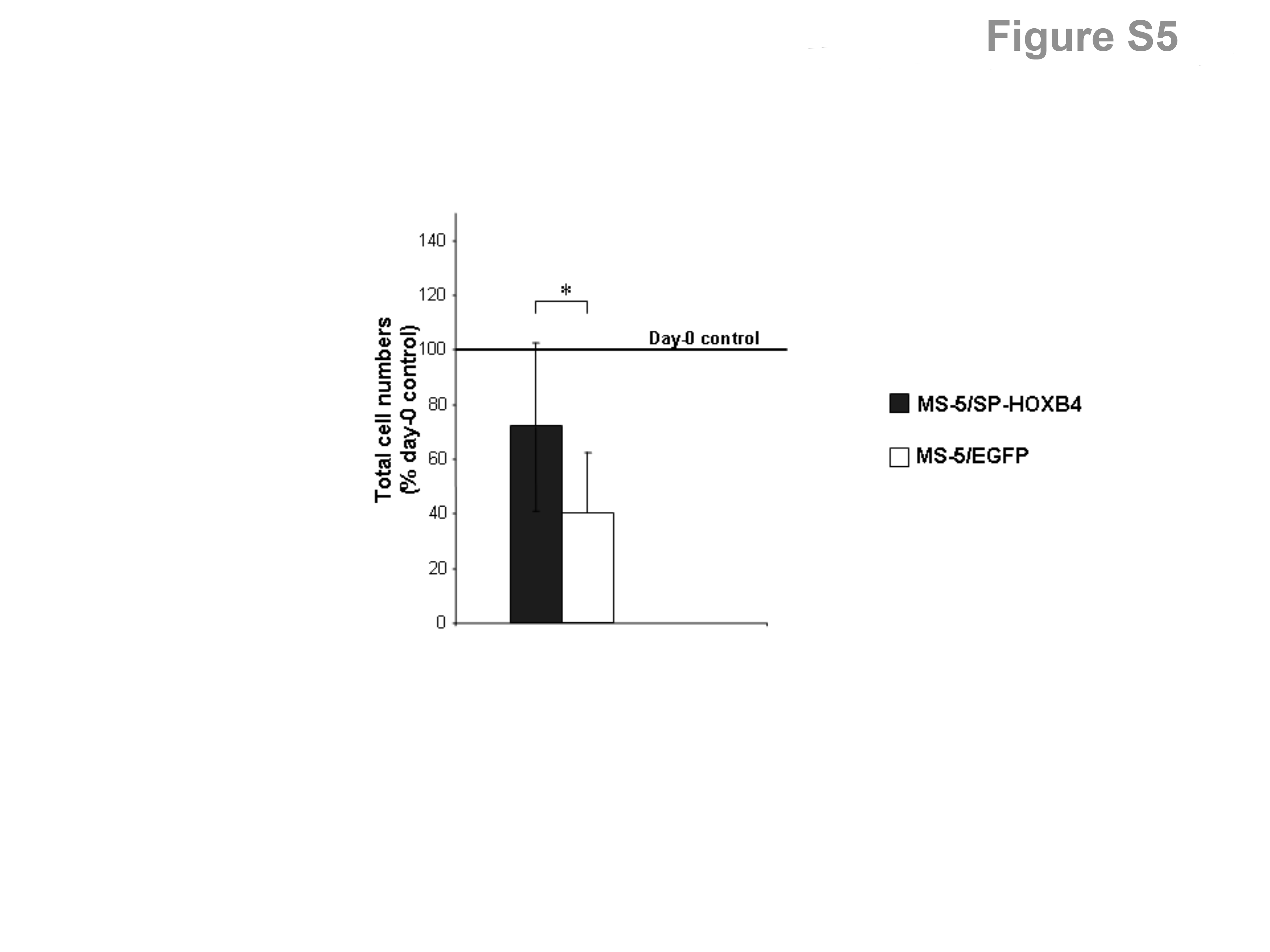

Supplement: Figure S5 — Fold increase of total cells in NK condition. Total cells (NK cells and non-NK cells) were derived from the primary 2-week co-cultures of hEB-derived cells with either MS-5/SP-HOXB4 or MS-5/EGFP control and then cultured under NK-cell differentiation condition for three weeks. Total cells were then numbered. Bar represents fold amplification relative to day-0 control (un-co-cultured hEBs) (designated as 100%) (n = 5, *p<0,05). (TIF) [file pone.0039514.s005.tif]
