# Supplementary material for: The HOXB4 Homeoprotein Promotes the Ex Vivo Enrichment of Functional Human Embryonic Stem Cell-Derived NK Cells
Source: PLoS One. 2012 Jun 27;7(6):e39514. doi: 10.1371/journal.pone.0039514 (PMC3384663; doi:10.1371/journal.pone.0039514)
Supplement: Table S1 — Primer sequences and access numbers (DOCX) [file pone.0039514.s006.docx]

| **Table S1** | | | |
| --- | --- | --- | --- |
| **Genes** | **Access numbers** | **Forward primers (5’–3’)** | **Reverse primers (5’–3’)** |
| ***HPRT*** | NM_000194.2 | TAATTGGTGGAGATGATCTCTCAAC | TGCCTGACCAAGGAAAAGC |
| ***MYC*** | NM_002467.4 | ACTCTGAGGAGGAACAAGAA | TGGAGACGTGGCACCTCTT |
| ***AML1/RUNX1*** | NM_1122607.1 | tcggctgagctgagaaatg | gtgatggtcagagtgaagctttt |
| ***GATA2*** | NM_032638.4 | AAGGCTCGTTCCTGTTCAGA | GGCATTGCACAGGTAGTGG |
| ***SCL/TAL-1*** | NM_1048166.1 | AACGCCAACTGGAGATTTCA | TTCTCGACCAGGATCAAAGC |
| ***BRCA2*** | NM_000059.3 | GCGCGGTTTTTGTCAGCTTA | TGGTCCTAAATCTGCTTTGTTGC |
| ***CASP8*** | NM_012115.2 | GAAATGAAAGCCCACCTCAA | GCTGAATTTGGTAAAAACACATCTT |
| ***EZH2*** | NM_004456.3 | TGGTCTCCCCTACAGCAGAA | TCATCTCCCATATAAGGAATGTTATG |
| ***GNL3*** | NM_014366.4 | CGAAGTCCAGCAAGTATTGAAG | TTCCTGTAGCCTGGGACAGT |
| ***HBP1*** | NM_014366.4 | AATATACTCAGATGTATCCAGGGAAAG | TTCCACCTGTCACCAAGGAA |
| ***HDAC2*** | NM_001527.2 | CAGATCGTGTAATGACGGTATCA | CCTTTTCCAGCACCAATATCC |
| ***IGFBP2*** | NM_000597.2 | GGTGGCAAGCATCACCTT | TCCTGTTGGCAGGGAGTC |
| ***IKZF*** | NM_006060.2 | CCTTCCGGGCACACTGTA | TCTCTCTGATCCTATCTTGCACA |
| ***YPEL5*** | NM_016061.1 | CACATCTCCACTCGTTTCACAGG | CGCTGTACTGCAGGTTAACTACC |
